# PAD-mediated citrullination is a novel candidate diagnostic marker and druggable target for HPV-associated cervical cancer

**DOI:** 10.3389/fcimb.2024.1359367

**Published:** 2024-03-11

**Authors:** Camilla Albano, Matteo Biolatti, Jasenka Mazibrada, Selina Pasquero, Francesca Gugliesi, Irene Lo Cigno, Federica Calati, Greta Bajetto, Giuseppe Riva, Gloria Griffante, Santo Landolfo, Marisa Gariglio, Marco De Andrea, Valentina Dell’Oste

**Affiliations:** ^1^ Department of Public Health and Pediatric Sciences, University of Turin, Turin, Italy; ^2^ Department of Cellular Pathology, The Cotman Centre Norfolk and Norwich University Hospital, Norwich, United Kingdom; ^3^ Department of Translational Medicine, University of Eastern Piedmont, Novara, Italy; ^4^ Center for Translational Research on Autoimmune and Allergic Disease-CAAD, Novara, Italy; ^5^ Department of Surgical Sciences, University of Turin, Turin, Italy; ^6^ IIGM Foundation – Italian Institute for Genomic Medicine, Turin, Italy; ^7^ Candiolo Cancer Institute, FPO-IRCCS, Turin, Italy

**Keywords:** human papillomaviruses, cervical cancer, citrullination, peptidylarginine deiminases, BB-cl-Amidine

## Abstract

Citrullination is an emerging post-translational modification catalyzed by peptidyl-arginine deiminases (PADs) that convert peptidyl-arginine into peptidyl-citrulline. In humans, the PAD family consists of five isozymes (PADs 1-4, 6) involved in multiple diseases, including cancer. Given that high-risk (hr) human papillomaviruses (HPVs) are the etiological agents of cervical cancer, in this study, we sought to determine whether PAD-mediated protein citrullination would play a functional role in the HPV-driven transformation of epithelial cells. Here we show that both total protein citrullination and PAD4 expression levels are significantly associated with cervical cancer progression. Specifically, epithelial immunostaining for PAD4 revealed an increasingly higher histoscore from low-grade (CIN1) to high-grade (CIN2, CIN3) cervical intraepithelial neoplasia, and invasive squamous cell carcinoma (SCC) lesions, raising the attractive possibility that PAD4 may be used as tumor staging markers. Furthermore, taking advantage of the epidermoid cervical cancer cell line CaSki, which harbors multiple copies of the integrated HPV16 genome, we show that the expression of E6 and E7 HPV oncoproteins is impaired by treatment with the pharmacological pan-PAD inhibitor BB-Cl-amidine. Consistently, p53 and p21, two targets of HPV oncoproteins, are upregulated by the PAD inhibitor, which undergoes cell growth arrest and apoptosis. Altogether, these findings highlight a novel mechanism by which hrHPVs alter host regulatory pathways involved in cell cycle and survival to gain viral fitness, raising the possibility that PADs may represent an attractive target for developing novel host-targeting antivirals effective in preventing cervical cancer progression.

## 1 Introduction

Human papillomaviruses (HPVs) are a major cause of human cancer, especially cervical cancer (Schiffman et al., 2016; McBride, 2022). Indeed, high-risk (hr) HPVs, in particular HPV16, are the etiological agents of almost 80% of all cervical cancer cases (de Villiers, 2013; Van Doorslaer et al., 2017). The development of HPV-associated cancers relies on the expression of two oncoproteins, E6 and E7, which are the only viral gene products consistently found in these tumors (Hoppe-Seyler et al., 2018; Scarth et al., 2021). Transformative capabilities have been demonstrated also for the HPV16 E5 oncoprotein. Nevertheless, the precise molecular mechanisms underlying its activity remain poorly understood. Unlike E6 and E7, the integration of episomal HPV DNA into the cellular genome results in the loss of the E5 open reading frame. Recent studies highlighted that E5 influences the initial phases of the transformation process rather than the later steps of malignant progression. It accomplishes this by modulating cellular processes like proliferation, differentiation, apoptosis, and energy metabolism through interactions with cell growth factor receptors and other cellular proteins (Gutierrez-Xicotencatl et al., 2021). As E6 and E7 do not possess intrinsic enzymatic activities, their transforming activity is thought to be predominantly exerted through direct or indirect interactions with cellular proteins, which ultimately favors the formation of the tumor environment (Mittal and Banks, 2017; Rasi Bonab et al., 2021). Recent evidence has shown that besides acting through the tumor suppressors p53 and retinoblastoma protein (pRb) (Vats et al., 2021), respectively, E6 and E7 can drive tumorigenesis through chromatin remodeling by altering the expression or the enzymatic activity of several epigenetic modifiers, such as histone deacetylases, histone demethylases, histone acetyltransferases, and histone methyltransferases (Durzynska et al., 2017; Burley et al., 2020). Concomitantly, HPVs have evolved strategies to subvert antiviral immunity and hamper cancer immunosurveillance, thereby favoring carcinogenesis (Zhou et al., 2019; Lo Cigno et al., 2020a, 2023; Girone et al., 2023). Even though the widespread implementation of vaccines has clear potential (Yousefi et al., 2021), no effective strategy for the treatment of low- or high-grade cervical lesions other than surgery is currently available (Zheng et al., 2022).

Citrullination, also called deimination, is a post-translational conversion of peptidyl-arginine to the non-genetically encoded amino acid peptidyl-citrulline, catalyzed by Ca^2+^-dependent, phylogenetically conserved, peptidyl-arginine deiminase (PAD) family of enzymes (Gudmann et al., 2015; Mondal and Thompson, 2021; Yu and Proost, 2022). In humans, the PAD family is composed of five highly-homologous isozymes (i.e., PADs 1-4 and 6), with different tissue-specific expression and substrate specificities (Bicker and Thompson, 2013; Witalison et al., 2015). Citrullination is particularly relevant to many human diseases, such as rheumatoid arthritis (Darrah and Andrade, 2018; Alghamdi and Redwan, 2021; Catrina et al., 2021), systemic lupus erythematosus (Singh et al., 2011), Alzheimer’s disease (Ishigami and Maruyama, 2010), Parkinson’s disease (Sancandi et al., 2020), and multiple sclerosis (Tu et al., 2016; Bruggeman et al., 2021). Higher expression of PAD genes has also been observed in various malignant tumors (Yuzhalin, 2019; Beato and Sharma, 2020; Zhu et al., 2021), suggesting their involvement in cancer pathogenesis.

A direct correlation between citrullination and viral infections has only recently emerged (Struyf et al., 2009; Muraro et al., 2018; Casanova et al., 2020). In this context, our group has recently unveiled the role of citrullination in promoting human cytomegalovirus (HCMV) and herpes simplex virus 1 (HSV-1) infection through the deimination of several cellular proteins, which promotes viral fitness (Griffante et al., 2021; Pasquero et al., 2023).

PADs are also involved in human epidermal keratinization and morphogenesis as well as skin tumorigenesis, processes closely linked to HPV transformation (Ying et al., 2009). Indeed, PAD2 overexpression in transgenic mice resulted in spontaneous skin neoplasia (Mohanan et al., 2017), and antibodies to citrullinated HPV-47 E2_345–362_ protein were found in patients affected by rheumatoid arthritis (Shi et al., 2008). Finally, *PADI4* levels were found to be significantly increased in the blood of patients with cervical cancer (Chang et al., 2009). However, how HPV induces protein citrullination in the host and whether citrullinated proteins can support viral replication in the host is currently unknown.

To gain more insights into the mechanisms of citrullination favoring disease progression and to identify more effective druggable targets and suitable biomarkers, the present study aimed to define the impact of PAD-mediated citrullination on HPV transformation. Our results reveal a significant association between PAD4 expression and cervical cancer progression. Accordingly, the pan-PAD-inhibitor BB-Cl-amidine (Ledet et al., 2018) downregulates the expression of HPV16 E6/E7, indicating that the process driven by PADs could be involved in HPV pathogenesis.

## 2 Materials and methods

### 2.1 Patients’ samples and data

Tissue sections were obtained from 100 formalin-fixed paraffin-embedded (FFPE) blocks, previously collected from surgically treated cervical lesions, and stored in the Norwich University Hospital material archives. The cervical lesions were obtained from high-risk HPV-positive women with abnormal cytology on cervical screening. In cases of high-grade (HG) cervical intraepithelial neoplasia (CIN)—at different stages (see below)—or invasive squamous cell carcinoma (SCC), tumor specimens were obtained by large loop excision of the transformation zone (LLETZ) or by hysterectomy. CIN is classified on a scale from one to three. CIN1 refers to abnormal cells affecting about one-third of the thickness of the epithelium, whereas CIN2 and 3 define abnormal cells present in one- to two-thirds or more than two-thirds of the epithelium, respectively (**Table 1**).

**Table 1:** Patient characteristics.

Lesions	Age (years)	Menopause	hrHPVs		p16		Follow-up (After 2 years)
			Positive	Negative	Positive	Negative	
NILM (n=20)	Mean=39.4 Range=22-83	3/20 (15%)	9/17 (52.9%) HPV16: 2/17 (11.8%) HPV18: 3/17 (17.6%) Others: 4/17 (23.5%)	8/17 (47.1%)	2/19 (10.5%)	17/19 (89.5%)	N.a.
CIN1 (n=20)	Mean=37.5 Range=25-62	2/19 (10.5%)	20/20 (100%) HPV16: 2/20 (10%) HPV18: 2/20 (10%) Others: 16/20 (80%)	0/20 (0%)	4/20 (20%)	16/20 (80%)	2/18 (11.1%) hrHPV persistence (no CIN)
CIN2 (n=20)	Mean=31 Range=24-44	0/20 (0%)	20/20 (100%) HPV16: 4/20 (20%) HPV18: 1/20 (5%) Others: 15/20 (75%)	0/20 (0%)	18/20 (90%)	2/20 (10%)	2/20 (10%) hrHPV persistence (no CIN)
CIN3 (n=20)	Mean=34.3 Range=24-53	2/20 (10%)	20/20 (100%) HPV16: 7/20 (35%) HPV18: 0/20 (0%) Others: 13/20 (65%)	0/20 (0%)	20/20 (100%)	0/20 (0%)	19/19 (100%) hrHPV negative
SCC (n=20)	Mean=42.8 Range=25-63	5/18 (27.8%)	18/18 (100%) HPV16: 6/18 (33.3%) HPV18: 4/18 (22.2%) Others: 8/18 (44.4%)	0/18 (0%)	20/20 (100%)	0/20 (0%)	1/14 (7.1%) Recurrence after 2 years

All procedures were performed in accordance with the ethical standards of the institutional Research Committee and with the 1964 Helsinki Declaration and its later amendments or comparable ethical standards.

All human tissues derived from biopsied cervical lesions were classified according to the 8^th^ Edition of the American Joint Committee on Cancer (AJCC-TNM) Staging Manual (TNM8) (Brierley et al., 2017).

Hematoxylin and eosin (H&E)-stained slides were reviewed to confirm the diagnosis and to assess the cytological and histomorphological features of each specimen.

### 2.2 HPV detection

HPV detection was performed using the cobas^®^ HPV Test (Roche Molecular Systems). Positive results were subcategorized into HPV16, HPV18, and other hrHPV types. p16 was used as a surrogate for hrHPV infection (**Table 1**).

### 2.3 Immunohistochemistry

Serial 5-μm sections from FFPE tissues were processed using the automated immunostainer Leica Bond III (Leica Biosystems). The primary antibodies employed are reported in **Supplementary Table S1**. Immunohistochemical expression of PAD4, PAD2, and anti-citrulline was evaluated by histo (H) score. For each histological section, the staining intensity was scored as 0 (negative), 1 (weak), 2 (moderate), and 3 (strong). The H-score was calculated by multiplying the intensity score (0-3) and the percentage of positive cells (0-100%), with a maximum of 300.

### 2.4 Cell cultures

CaSki cells (ATCC CRL-1550™) were cultured in RPMI-1640 medium and HeLa cells (ATCC CCL-2™) in Dulbecco’s Modified Eagle’s medium, both supplemented with 10% heat-inactivated fetal bovine serum (FBS), 2 mM glutamine, 1 mM sodium pyruvate, 100 U/mL penicillin, and 100 µg/mL streptomycin sulfate (Sigma-Aldrich). HPV-negative normal oral keratinocytes (NOKs) were kindly provided by Frank Rösl (Germany) and were cultured as previously described (Yang et al., 2019).

### 2.5 Transfection

CaSki and HeLa cells were transiently transfected with small interfering RNAs (siRNAs) using a Neon™ Transfection System (Life Technologies) according to the manufacturer’s instructions (1005 V, 35 ms pulse width, two impulses). The following siRNAs were used: control siRNA (siCTRL; 1027292) was purchased from Qiagen; siRNAs against HPV16 E6/E7#1,HPV16 E6/E7#2, HPV18 E6/E7#1, and HPV18 E6/E7#2 were synthesized by Eurofins Genomics. The indicated siRNAs were previously characterized (Lo Cigno et al., 2020b), and the specific sequences are reported in **Supplementary Table S2**.

### 2.6 Compounds

BB-Cl-amidine (BB-Cl-A; HY-111347A) (Ledet et al., 2018) was purchased by MedChemExpress and dissolved in dimethyl sulfoxide (DMSO; Sigma-Aldrich) at stock concentrations of 25 mM.

### 2.7 Cell viability assay

CaSki viability upon exposure to increasing concentrations of BB-Cl-A or DMSO was determined by 3-(4,5-dimethylthiazol-2-yl)-2,5diphenyltetrazolium bromide (MTT) method (Toscani et al., 2021).

### 2.8 Quantitative nucleic acid analysis

Total RNA was extracted using TRI Reagent (Sigma-Aldrich), and 1 μg was retrotranscribed using the Revert-Aid H-Minus First Strand cDNA Synthesis Kit (Thermo Fisher Scientific), according to the manufacturer’s instructions. Comparison of mRNA expression between samples was performed by SYBR green-based RT-qPCR using Mx3000P apparatus (Stratagene). The housekeeping gene glyceraldehyde 3-phosphate dehydrogenase (GAPDH) was used to normalize for variation in cDNA levels. The primer sequences used are reported in **Supplementary Table S2**.

### 2.9 Western blot analysis

Protein extracts were prepared in RIPA buffer and subjected to immunoblotting. The primary antibodies used are reported in **Supplementary Table S1**. Scanning densitometry was performed using Image Lab (version 6.0.1; Bio-Rad).

### 2.10 Detection of citrullination with rhodamine–phenylglyoxal

Equal amounts of protein were diluted with 80% trichloroacetic acid and incubated with Rh–PG (final concentration 0.1 mM; Cayman Chemical) for 30 min. The reaction was quenched with 100 mM L-citrulline (Sigma-Aldrich), centrifuged at 21,100×g for 10 min, washed with ice-cold acetone, resuspended in PBS supplemented with L-arginine and analyzed through gel electrophoresis. Gels were imaged (excitation = 532 nm, emission = 580 nm) using a ChemiDoc MP Imaging System (Bio-Rad Laboratories), stained with brilliant blue G-colloidal solution (Sigma-Aldrich).

### 2.11 Cell cycle analysis

CaSki cells were seeded in 6-well plates and treated with BB-Cl-A (3 μM) for 24 or 48 h. After treatment, cells were pelleted down and fixed with 70% methanol for 30 min at 4°C. After washing with PBS twice, cells were incubated with a DNA staining solution consisting of propidium iodide (PI; Sigma-Aldrich) and RNase (Merck Millipore) for 15 min at 37°C in the dark. The proportion of cells in each phase of the cell cycle was determined by DNA content stained with PI using a BD FACSCanto II flow cytometer (BD Biosciences). Data obtained were analyzed with FlowJo software (BD Biosciences).

### 2.12 Apoptosis detection

To distinguish apoptotic from necrotic cells, double staining was performed for exposed phosphatidylserine and PI exclusion using the annexin V-FITC Apoptosis Detection Kit (Calbiochem). Experiments were performed according to the manufacturer’s instructions. Briefly, CaSki cells were seeded in 6-well plates and treated with BB-Cl-A (3 μM) for 24 or 48 h. After treatment, cells were washed with PBS twice, trypsinized, and then resuspended in a binding buffer (10 mM HEPES/NaOH, pH 7.4, 140 mM NaCl, 2.5 mM CaCl_2_). Annexin V-FITC was added to a final concentration of 100 ng/mL, and the cells were incubated in the dark for 10 min, then washed again in PBS, and resuspended in 300 μL of the binding buffer. In total, 40 μg/mL of PI was added to each sample before the flow cytometric analyses. Cells were analyzed using a BD FACSCanto II flow cytometer (BD Biosciences). Data obtained were analyzed with FlowJo software (BD Biosciences). Unstained cells and cells only stained with annexin V-FITC or PI were used as controls to establish compensation and quadrants. Cells were gated according to their light-scatter properties to exclude cell debris.

### 2.13 Statistical analysis

All statistical tests were performed using GraphPad Prism version 7.04 for Windows (GraphPad Software). Data are presented as means ± SEM or medians ± interquartile. Means were compared using an unpaired t-test, meanwhile, medians were compared using a one-way analysis of variance (ANOVA) with Bonferroni’s post-tests. The two-tailed Pearson correlation was employed to assess th e correlation between PAD4 and citrulline expression in immunohistochemistry. Differences were considered statistically significant at *P* < 0.05 (*, *P* < 0.05; **, *P* < 0.01; ***, *P* < 0.001).

## 3 Results

### 3.1 Enhanced total protein citrullination and PAD4 expression in cervical intraepithelial neoplasia and invasive squamous cell carcinoma

To evaluate the role of citrullination in the context of hrHPV-related lesions, we assessed the citrullination profiles of FFPE biopsies from samples negative for intraepithelial lesion or malignancy (NILM) *vs.* cervical intraepithelial neoplasia (CIN) at different stages — from CIN (CIN1 > CIN2 > CIN3, see Materials and Methods) to invasive SCC. Information about patients’ age, menopausal status (yes/no), and follow-up is presented in **Table 1**. The mean age of the cohort was 47 years, ranging from 22 to 63 years, with 12.2% of women in a menopausal state. HrHPV and p16 positivity are also reported and agree with previous findings (Kombe Kombe et al., 2021; Shafique et al., 2023). A recurrence of carcinoma has been reported after 2 years in 1 out of 14 SCC cases with available follow-up data.

**Figure 1:**
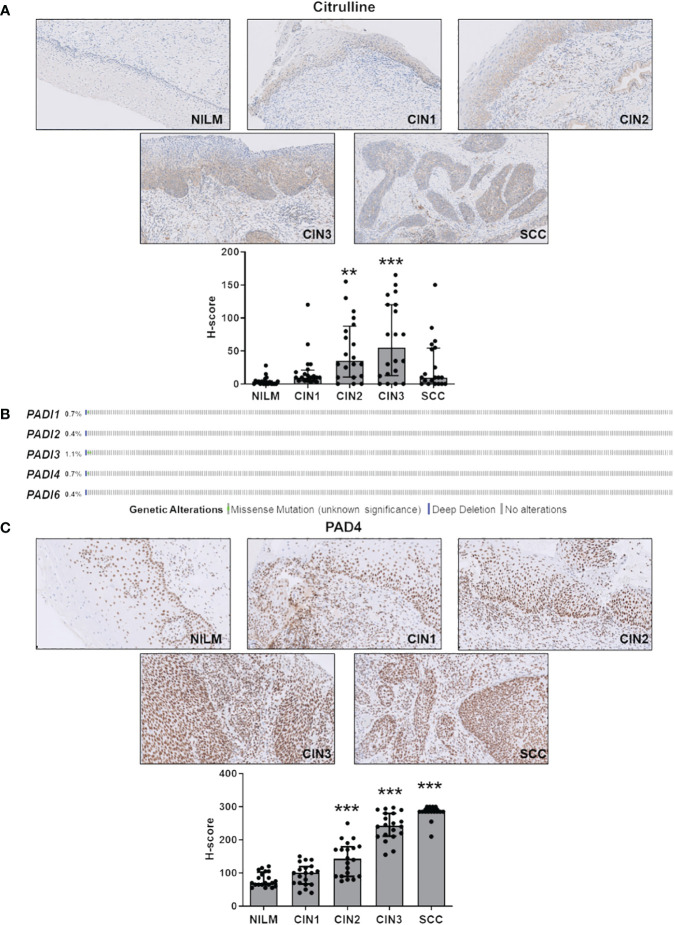
Citrulline and PAD4 expression in CINs and SCC. **(A)** Citrulline immunohistochemical photomicrographs of representative biopsies of mucosa negative for intraepithelial lesion or malignancy (NILM) and different stages of cervical carcinogenesis (CIN1, CIN2, CIN3, and SCC). Hematoxylin was used for counterstaining. Original magnification: 20X. In the lower histogram, the collective presentation of quantified anti-citrulline IHC score in NILM (n=20), CIN1 (n=20), CIN2 (n=20), CIN3 (n=20), and SCC (n=20). The H-score median and interquartile range for each group are shown. Medians were compared using a one-way analysis of variance (ANOVA) with Bonferroni’s post-tests. *P* < 0.05 (**, *P* < 0.01; ***, *P* < 0.001). **(B)** TCGA-curated clinical data set of cervical squamous cell carcinoma (n=278) was assessed for samples harboring genomic *PADI* gene loss (solid blue) and/or missense mutations (green dot). **(C)** PAD4 immunohistochemical photomicrographs of representative biopsies of normal mucosa and different stages of cervical carcinogenesis as above. Hematoxylin was used for counterstaining. Original magnification: 20X. In the lower histogram, the collective presentation of quantified PAD4 IHC scores in NILM (n=20), CIN1 (n=20), CIN2 (n=20), CIN3 (n=20), and SCC (n=20). The H-score median and interquartile range for each group are shown. Medians were compared using a one-way analysis of variance (ANOVA) with Bonferroni’s post-tests. *P* < 0.05 (**, *P* < 0.01; ***, *P* < 0.001).

As shown in **Figure 1A** and **Supplementary Table S3**, disease progression was paralleled by a significant increase in total protein citrullination levels in cells with aberrant proliferative capacity, and this effect is more pronounced in CIN2 and CIN3 lesions, while in the SCC group, there was significant variability in citrulline staining. Specifically, keratinocytes with high and abnormal levels of citrullinated proteins in the cytoplasm were localized throughout the mucosal layers, whereas in the normal epithelium, they were predominantly found in the basal and parabasal layers (**Supplementary Table S3**).

Since PADs are the enzymes catalyzing citrullination, we next sought to determine their expression according to tumor stage. To rule out bias due to allelic loss in tumors, we first examined PAD expression in a squamous cervical carcinoma data set curated by TCGA (Gao et al., 2013; Ciriello et al., 2015). We found the PAD genomes rarely mutated—less than 1.2% out of 278 tumors, considering all PAD isoforms (**Figure 1B**)—indicating that loss of PAD expression is a rare event.

Next, we assessed cervical cancer *vs.* NILM for protein expression levels of PADs 2 and 4, the two PAD isozymes most broadly expressed in human cancer (Yuzhalin, 2019; Sarnik and Makowska, 2022). In NILM, PAD4 expression was limited to the nuclei in basal and parabasal layers of the squamous epithelium and in sparse stromal and inflammatory cells within the stromal compartment (**Figure 1C**). On the other hand, PAD4 staining of both the atypical squamous epithelium and the stromal compartment significantly increased as the cancer progressed to more advanced stages (**Figure 1C**). Interestingly, this increase was significantly correlated with enhanced citrullinated protein expression levels in CIN2 and CIN3 lesions but not CIN1 and SCC (**Supplementary Table S3**). No signal was observed in negative controls that were incubated with either the primary or secondary antibody alone (data not shown). Of note, koilocytes in low-grade CIN exhibited a very weak PAD4 immunoreactivity in comparison with high-grade lesions, which suggests differential expression of this marker according to the integration status of HPV in squamous cells. Moreover, mature squamous cells in the upper epithelial layers were PAD4 negative, which implies that PAD4 expression can be detected only in immature squamous phenotypes.

Collectively, these results argue in favor of PAD4-mediated citrullination as a critical event in cervical tumorigenesis. In contrast, PAD2, whose expression is restricted to the glandular epithelium and absent in the squamous epithelium in both normal mucosa and hrHPV-related lesions (**Supplementary Figure S1**), does not seem to be involved in disease progression.

### 3.2 Impact of hrHPV E6/E7 on PAD expression profile

**Figure 2:**
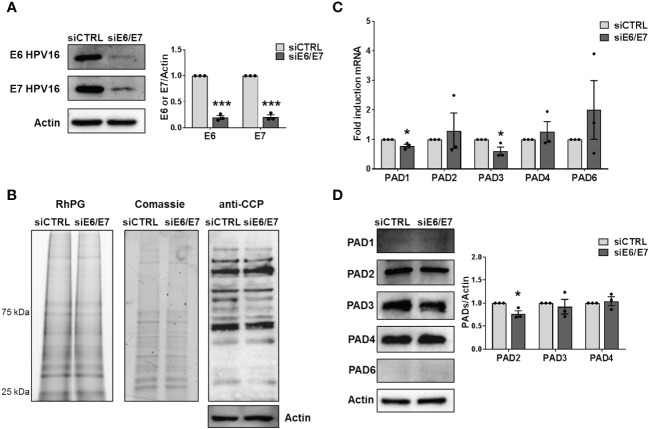
Citrullination analysis. **(A)** CaSki cells were transfected with siRNA E6/E7 or siRNA CTRL and processed at 72 h post-transfection. The efficiency of E6 and E7 protein depletion was determined by immunoblotting using antibodies against E6 and E7 and Actin as control. Values are expressed as means ± SEM (error bars) of three independent experiments. *P* < 0.05 (*, *P* < 0.05; **, *P* < 0.01; ***, *P* < 0.001, unpaired t-test). **(B)** Detection of citrullinated proteins in lysates obtained from siRNA E6/E7- or siRNA CTRL-transfected CaSki cells at 72 h post-transfection. *Left panels:* lysates were exposed to an Rh-PG citrulline-specific probe and subjected to gel electrophoresis to detect total proteins. Equal loading was assessed by Coomassie blue staining. *Right panel:* the indicated samples were analyzed by immunoblotting, and citrullinated proteins were detected using an anti-CCP antibody and Actin as a loading control. **(C)** mRNA expression levels of *PADI* isoforms by RT-qPCR of siRNA E6/E7- or siRNA CTRL-transfected CaSki cells were normalized to the housekeeping gene GAPDH and expressed as mean fold change ± SEM over siRNA CTRL. *P* < 0.05 (*, *P* < 0.05; ***, *P* < 0.001, unpaired t-test). **(D)** Western blot analysis of protein lysates from siRNA E6/E7- or siRNA CTRL-transfected CaSki cells using antibodies against PAD1, PAD2, PAD3, PAD4, PAD6, or Actin. One representative blot and densitometric analysis relative to three independent experiments are shown. Values are expressed as mean fold change ± SEM normalized to Actin. *P* < 0.05 (*, *P* < 0.05; ***, *P* < 0.001, unpaired t-test).

To investigate whether HPV trigger PAD-mediated citrullination to promote viral fitness, we first evaluate the protein citrullination profile in CaSki cells, which contain about 600 copies of HPV16 genomes per cell (Mincheva et al., 1987). Protein lysates obtained from either siRNA E6/E7- or siRNA CTRL-transfected CaSki were exposed to the citrulline-specific probe Rh-PG (Bicker et al., 2012). Even though the expression of the viral proteins was almost completely suppressed upon gene silencing (**Figure 2A**), as demonstrated by immunoblotting with antibodies raised against HPV E6 and E7, we did not notice any significant differences between the citrullination profiles of cells lacking E6 and E7 (siE6/E7) and their controls (siCTRL) (**Figure 2B**, left panel), neither the overall amount of proteins was modified (**Figure 2B**, middle panel). The same results were obtained when membranes were probed with an anti-cyclic citrullinated peptide (CCP) antibody (**Figure 2B**, right panel). We detected the same citrullination pattern in HeLa cells transfected with siRNA E6/E7 or siRNA CTRL (**Supplementary Figures S2A, B**), confirming that the overall citrullination profile is not markedly affected by the absence of hrHPV E6/E7. Furthermore, RT-qPCR analysis of RNAs from the same cells revealed that *PADI* genes were all expressed in CaSki and HeLa, but not profoundly modulated by E6 and E7 compared to the controls (**Figure 2C**, **Supplementary Figure S2C**). When we analyzed PAD protein expression, we only observed a slight downregulation of PAD2 in CaSki and HeLa cells lacking E6 and E7 (24% and 22%, respectively), while PAD3 and PAD4 were not significantly modulated. Other PAD isoforms (PAD1 and 6) were expressed at undetectable levels in CaSki and HeLa cells and did not vary upon E6/7 gene silencing (**Figure 2D**, **Supplementary Figure S2D**).

### 3.3 The pan-PAD inhibitor BB-Cl-amidine subverts hrHPV E6/E7-related pathways

**Figure 3:**
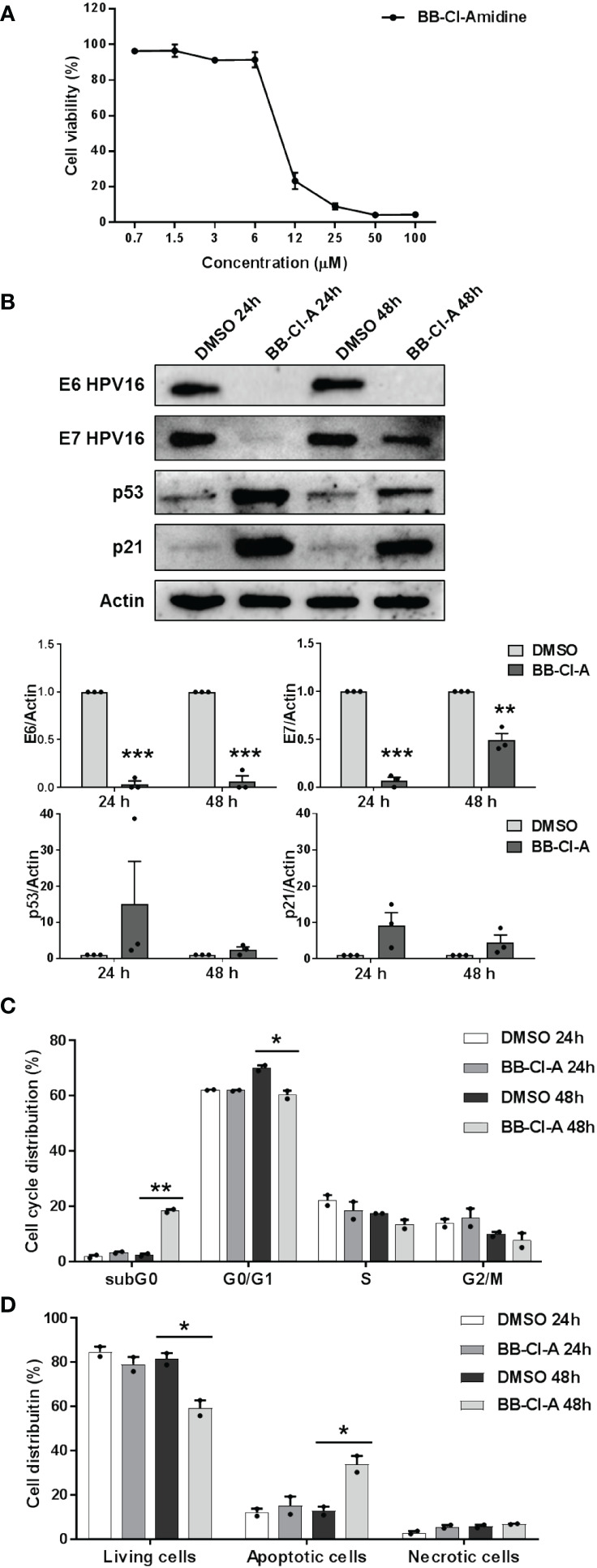
Antiviral activity of the pan-PAD inhibitor BB-Cl-amidine against HPV transformation. **(A)** CaSki cells were treated with increasing concentrations of the cell-permeable pan-PAD inhibitor BB-Cl-amidine (BB-Cl-A). At 48 h post-treatment (hpt), the number of viable cells was determined for each BB-Cl-A concentration by MTT. Values are expressed as means ± SEM (error bars) of three independent experiments. **(B)** Protein lysates from CaSki cells treated with 3 µM BB-Cl-A were subjected to immunoblotting using antibodies against E6, E7, p53, p21, or Actin. One representative blot and predictive densitometric analysis relative to three independent experiments are shown. Values are expressed as means ± SEM. Differences were considered statistically significant for *P* < 0.05 (*, *P* < 0.05; **, *P* < 0.01; ***, *P* < 0.001, unpaired t-test). **(C)** Cell cycle analysis of BB-Cl-A-treated cells by flow cytometry. CaSki cells were treated with 3 µM of BB-Cl-A or DMSO for 24 and 48 (h) Values were plotted as the percentage of cell distribution across the different cell cycle phases (SubG_0_, G_0_/G_1_, S, and G_2_/M). Each bar represents the mean of data obtained from two independent experiments. Differences were considered statistically significant for *P* < 0.05 (*, *P* < 0.05; **, *P* < 0.01; ***, *P* < 0.001, unpaired t-test). **(D)** CaSki cells were treated with 3 µM of BB-Cl-A or with equal volumes of DMSO. After 24 and 48 h, cells were processed for Annexin V/propidium iodide (PI) flow cytometric analysis. Annexin−/PI− cells indicated living cells, Annexin+/PI− apoptotic cells, and Annexin+/PI+ necrotic cells. Values were plotted as the percentage of cell distribution across the two different conditions. Fold changes were calculated after the normalization of BB-Cl-A *vs.* DMSO-treated cells. Differences were considered statistically significant for *P* < 0.05 (*, *P* < 0.05, unpaired t-test).

To conclusively elucidate the impact of PAD-mediated citrullination on hrHPV pathogenesis, we took advantage of the cell-permeable pan-PAD inhibitor BB-Cl-amidine (BB-Cl-A). However, before assessing BB-Cl-A activity on CaSki cells, we performed a standard MTT viability assay to rule out the possibility that the drug may have cytotoxic effects. Indeed, our screening indicated that the cytotoxicity of BB-Cl-A was low or undetectable at concentrations of up to 3 µM, as ~90% of the cells were viable after 48 h of treatment (**Figure 3A**).

We then evaluated E6 and E7 protein expression levels in CaSki cells treated or not with BB-Cl-A. As shown in **Figure 3B**, we observed a dramatic downregulation of both E6 and E7 expression in BB-Cl-A-treated cells, with a more pronounced effect after 24 h. Conversely, an expected increase in p53 protein expression, a known target of E6 (Mittal and Banks, 2017), was detected at 24 and 48 h post-BB-Cl-A treatment (15 and 2.4 folds at 24 and 48 h *vs.* vehicle control, respectively). Fittingly, at the same time points, we also recorded a 9.2 (24 h) and 4.5 (48 h) fold increase of the cyclin-dependent kinase inhibitor p21 (Harris and Levine, 2005), a p53, and E7 target gene. Overall, these results demonstrate that the inhibitory activity of the BB-Cl-A compound against hrHPV E6 and E7 is associated with the upregulation of p53 and p21 expression. This effect is specific, as p53 and p21 were not modulated by BB-Cl-A in HPV-negative NOKs (**Supplementary Figure S3**).

Finally, since p53 target genes, such as p21, are involved in the regulation of cell cycle progression (Xiong et al., 1993), we sought to determine the effect of BB-Cl-A treatment on the cell cycle. Flow cytometry analyses showed that upon 48 h of treatment, BB-Cl-A significantly increased the population of CaSki cells in the sub-G_0_ phase, while decreasing in the G_0_-G_1_ phase, compared to control, indicating that PAD inhibition leads to cell cycle arrest (**Figure 3C**). To further strengthen this observation and given that apoptosis is one of the major mechanisms by which hosts evade viral infections, including HPV (Shimada et al., 2020; Gusho and Laimins, 2022), we assessed the ability of BB-Cl-A to drive cell-death pathways in CaSki cells by dual staining with annexin V and PI. As shown in **Figure 3D**, at 48 hpt (hours post-treatment), a significant increase in apoptosis (annexin V+/PI−) was detected in cells treated with BB-Cl-A compared to vehicle-treated cells (33.9% *vs.* 12.73%, respectively), while necrosis (annexin V+/PI+) barely occurred in every condition tested.

Altogether, our findings indicate that the inhibition of protein citrullination by BB-Cl-A halts cell cycle progression at the sub-G_0_ phase and drives CaSki cells to apoptosis, suggesting that BB Cl-A is a promising pharmacological agent to treat HPV-infected cells.

## 4 Discussion

In the present study, we report for the first time that citrullination plays a role in hrHPV pathogenesis in the context of cervical cancer. Indeed, we find a significant association between the overall citrullination pattern, PAD expression, and cervical cancer progression in a cohort of patients with different stages of cervical lesions. Accordingly, in an *in vitro* model of persistent hrHPV transformation - i.e. CaSki cells - the expression of E6 and E7 HPV oncoproteins is downregulated by the pan-PAD inhibitor BB-Cl-A, followed by robust upregulation of p53 and p21, the main targets of HPV oncoproteins. Conversely, total protein citrullination and PAD expression do not seem to be affected by E6 and E7 expression *in vitro*, in both CaSki and HeLa cells (**Supplementary Figure S4**).

Increasing evidence is emerging about the relevance of citrullination to human diseases. Indeed, besides the upregulation of PAD isozymes in many autoimmune disorders (Bicker and Thompson, 2013; Bruggeman et al., 2021), recent studies highlighted a modulation of citrullination in the context of viral infections (Griffante et al., 2021; Pasquero et al., 2022, 2022).

Our group has previously shown that HCMV infection induces citrullination in human fibroblasts and that PAD2, the isoform mainly induced upon infection, is essential for HCMV replication (Griffante et al., 2021). This might represent an alternative strategy for efficient inhibition of HCMV replication even in the presence of drug resistance mechanisms due to viral DNA polymerase mutations. Furthermore, we have recently found the same ability to exploit PAD-mediated citrullination in order to achieve enhanced viral growth in *in vitro* models of β-coronavirus infection, i.e., HCoV-OC43 and SARS-CoV-2 (Pasquero et al., 2022) and HSV-1 replication (Pasquero et al., 2023). Interestingly, we failed to observe a robust modulation of the citrullination profile or PAD expression in the context of hrHPV transformation *in vitro*, even upon silencing the E6 and E7 viral oncoproteins. This could be ascribed to an already mutated cellular phenotype due to HPV integration, with citrullination and PAD expression levels already saturated. As such, it is likely that citrullination was not altered *per se* but might influence cellular pathways linked to HPV-induced transformation. Fittingly, the pan-PAD inhibitor BB-Cl-A impairs E6 and E7 expression. As a consequence, p53 and p21, targeted by E6 and E7, are restored, leading to a sub-G_0_ cell cycle block in cells treated with BB-Cl-A and an increased rate of apoptotic cells. The impact of BB-Cl-A is more evident at 24 h, while at 48 h, E7 protein levels increase, albeit remaining significantly downregulated compared to the control. This phenomenon may be attributed to various factors, such as feedback mechanisms, adaptive responses, protein turnover, or alternative regulatory pathways. However, determining these factors precisely extends beyond the scope of the current study. PAD inhibitors have already been successfully employed in preclinical and *in vitro* studies for various inflammatory conditions, such as arthritis, colitis, and sepsis, showing a good safety profile (Chumanevich et al., 2011; Willis et al., 2011). This represents an important aspect that supports the repurposing of these compounds as antivirals to counteract HPV-related carcinogenesis.

Our findings that citrullinated proteins and PAD4 are overexpressed in HPV-positive cervical cancers support the role of citrullination in HPV transformation, in agreement with the higher expression of *PADI4* genes found in various malignant tumor tissues, as well as in the blood of patients with some cancers (Chang et al., 2009; Yuzhalin, 2019). Interestingly, we showed that citrulline and PAD4 H-scores correlated significantly in CIN2 and CIN3, while in the carcinoma group, there was significant variability in the expression of citrullinated proteins. This observation is consistent with previous results obtained with lung cancer, demonstrating that citrullination was a less specific marker for the tumor (Baka et al., 2011). Alternatively, we could speculate that even though high levels of PAD4 are detectable in invasive cancer, we do not know whether the protein still retains its enzymatic activity. Interestingly, a single recurrence was observed in a carcinoma with low detectedcitrullinated proteins (H-score 10), suggesting a possible protective role of citrulline during cervical carcinogenesis. Variation in citrullinated protein levels may therefore be used as an adjunctive criterion to predict the biological behavior of tumor in addition to other clinical and surgical criteria (e.g. completeness of excision, stage, grade, age of patients, risk factors, etc). This warrants further studies, however, on a larger number of specimens.

Another evidence of citrullination involvement in HPV-driven carcinogenesis arises from the observation that p53 is strongly upregulated in BB-Cl-A-treated CaSki cells, in line with previous findings indicating that the expression of p53 target genes is reduced in cells overexpressing PAD4, resulting in the perturbation of the normal cell cycle (Li et al., 2008). Of note, PADs are also involved in human epidermal keratinization and morphogenesis, as well as in skin tumorigenesis, a process closely linked to HPV infection. For instance, differential expression of the four genes encoding PAD1, together with laminin-γ2 (LAMC 2), collagen type IV α1 (COL4A 1), and collagen type I α1 (COL1A 1), has been proposed to be a predictive biomarker of squamous cell carcinomas of the oral cavity and oropharynx (Chen et al., 2008). Finally, stromal CD66b^+^ neutrophils and myeloperoxidase/citrullinated histone H3 (MPO/H3Cit)-labeled neutrophil extracellular trap (NETs) have been recently identified as an independent prognostic factor for recurrence-free survival (RFS) in cervical cancer (Yan et al., 2021).

Surprisingly, our immunohistochemical analysis of cervical carcinoma tissue specimens found that PAD2, unlike PAD4, is not linked to cervical cancer progression and that its expression is restricted to the glandular epithelium of both normal mucosa and hrHPV-related lesions. This is somewhat surprising given that PAD2 overexpression in transgenic mice promotes skin neoplasia (McElwee et al., 2014; Mohanan et al., 2017). In addition, PAD2 was found to be overexpressed in patients with many types of tumor tissues, such as castration-resistance prostate cancer (CRPC) (Wang et al., 2017), invasive breast ductal carcinoma, cervical squamous cell carcinoma, colon adenocarcinoma, liver hepatocellular carcinoma, lung cancer, ovarian serous papillary adenocarcinoma, and papillary thyroid carcinoma samples (McElwee et al., 2012; Guo et al., 2017). However, and in good agreement with our results, downregulation of PAD2 is an early event in the pathogenesis of colorectal cancer associated with poor prognosis (Cantariño et al., 2016), suggesting that PAD expression is strictly dependent on the tumor microenvironment, while their tumorigenic effects and mechanisms are still controversial.

Overall, our findings provide a new paradigm of hrHPV-host interplay based on PAD-mediated citrullination that could ultimately be exploited for further diagnostic and therapeutic development to curb HPV transformation in infected patients.

## Data availability statement

The raw data supporting the conclusions of this article will be made available by the authors, without undue reservation.

## Ethics statement

This research was approved by the Cambridge East Research Ethics Committee (Ethics application ID: BAC.007.22; ref EE/19/0089) and adhered to the principles of the Declaration of Helsinki. The studies were conducted in accordance with the local legislation and institutional requirements. Written informed consent for participation was not required from the participants or the participants’ legal guardians/next of kin because The investigations were conducted in compliance with local legislation and institutional requirements. Written informed consent for participation was not required from the participants or the participants’ legal guardians/next of kin. The study, involving the use of paraffin-embedded material from existing records, was sanctioned by the Cambridge East Research Ethics Committee. In the UK, oral consent was deemed sufficient for cervical biopsies performed after abnormal HPV results.

## Author contributions

CA: Writing – original draft, Methodology, Investigation, Data curation, Conceptualization. MB: Writing – original draft, Supervision, Investigation, Funding acquisition, Data curation. JM: Writing – original draft, Methodology, Investigation, Data curation, Conceptualization. SP: Writing – review & editing, Investigation. FG: Writing – review & editing, Investigation, Funding acquisition. IC: Writing – review & editing, Supervision, Investigation. FC: Writing – review & editing, Methodology. GB: Writing – review & editing, Methodology. GG: Writing – review & editing, Investigation. GR: Writing – review & editing, Conceptualization. SL: Writing – review & editing. MG: Writing – review & editing, Project administration, Funding acquisition. MD: Writing – review & editing, Funding acquisition. VD: Writing – original draft, Supervision, Project administration, Funding acquisition, Data curation, Conceptualization.
