# Supplementary material for: PAD-mediated citrullination is a novel candidate diagnostic marker and druggable target for HPV-associated cervical cancer
Source: Front Cell Infect Microbiol. 2024 Mar 11;14:1359367. doi: 10.3389/fcimb.2024.1359367 (PMC10961408; doi:10.3389/fcimb.2024.1359367)
Supplement: Supplementary file 1 [file DataSheet_1.pdf]

Supplementary Material

1 Supplementary Figures and Tables

1.1 Supplementary Tables

TABLE S1. Antibodies used in this study.

Antigen	Species and isotype	Source	WB dilution	IHC dilution
PAD1	Rabbit polyclonal	ab24008<br>Abcam	1:500	
PAD2	Rabbit polyclonal	ROI002<br>Cosmo Bio	1:1000	1:2000
PAD3	Rabbit polyclonal	ab172959<br>Abcam	1:500	
PAD4	Rabbit polyclonal	ab128086<br>Abcam	1:500	1:2000
PAD6	Rabbit polyclonal	ab169416<br>Abcam	1:500	
citrulline	Mouse IgG_{2b}	clone 1D9<br>Cay30773<br>Cayman Chemical		1:2000
CCP	Rabbit polyclonal	orb10262<br>Biorbyt	1:500	
HPV16 E6	Rabbit polyclonal	GTX32686<br>GeneTex	1:500	
HPV16 E7	Rabbit polyclonal	GTX133411<br>GeneTex	1:500	
HPV18 E6	Rabbit polyclonal	GTX132687<br>GeneTex	1:500	
HPV18 E7	Rabbit polyclonal	GTX133412<br>GeneTex	1:500	
p53	Mouse IgG_{2a}	clone DO-1<br>sc-126<br>Santa Cruz Biotechnology	1:500	
p21	Mouse IgG_{2b}	clone CP74<br>P1484<br>Sigma-Aldrich	1:500	
Actin	Mouse IgG_{2b}	clone C4<br>MAB1501<br>Sigma-Aldrich	1:1000	

WB (Western blot); IHC (Immunohistochemistry).

TABLE S2. Oligonucleotide sequences.

**Oligonucleotide designation**	**Sequence (5' to 3')**
HPV16 E6E7#1 (siE6/E7) Fw	GACAGAGCCCAUUACAAUA
HPV16 E6E7#1 (siE6/E7) Rv	UAUUGUAAUGGGCUCUGUC
HPV16 E6E7#2 (siE6/E7) Fw	GCACACACGUAGACAUUCG
HPV16 E6E7#2 (siE6/E7) Rv	CGAAUGUCUACGUGUGUGC
HPV18 E6E7#1 (siE6/E7) Fw	CUCUGUGUAUGGAGACACAU
HPV18 E6E7#1 (siE6/E7) Rv	AUGUGUCUCCAUACACAGAG
HPV18 E6E7#2 (siE6/E7) Fw	CGAUGAAAUAGAUGGAGUU
HPV18 E6E7#2 (siE6/E7) Rv	AACUCCAUCUAUUUCAUCG
HPV18 E6E7#2 (siE6/E7) Fw	GCUAGUAGUAGAAAGCUCA
HPV18 E6E7#2 (siE6/E7) Rv	UGAGCUUUCUACUACUAGC
siCTRL (Qiagen, code 1027292)	N.a.
PADI1 Fw	TCCAGAGACCCTGAAGCTGT
PADI1 Rv	GTGCAGCTGTCCCTGAAGAT
PADI2 Fw	ACCTCCTCAGCCTCCCC
PADI2 Rv	CCTACCTCTGGACCGATGTC
PADI3 Fw	GCGTCCCATAGACCTCAAAC
PADI3 Rv	CAGAGAATCGTGCGTGTGTC
PADI4 Fw	CCTGTGGATTTCTTCTTGGC
PADI4 Rv	GGGCACCTTGACTCAGCTT
PADI6 Fw	CAAGGTATAGGCGTGCTGGT
PADI6 Rv	TCCTCCATACCTCCAAGGAA
GAPDH Fw	AACGTGTCAGTGGTGGACCTG
GAPDH Rv	AGTGGGTGTCGCTGTTGAAGT

TABLE S3. Immunoreactive scores of PAD4 and citrullinated proteins.

**Lesions**	**PAD4 (H-score)**	**Citrulline (H-score)**	**PAD4 *vs.* citrulline (*p*-value*)**
NILM (n=20)	Median=70 Range=55-120	Median=3 Range=0-28	0.5942
CIN1 (n=20)	Median=100 Range=40-150	Median=10 Range=3-120	0.1207

CIN2 (n=20)	Median=142.5 Range=75-250	Median =35 Range=0-155	0.0377
CIN3 (n=20)	Median=242.5 Range=155-294	Median=55 Range=0-165	0.0482
SCC (n=20)	Median=285 Range=210-300	Median=10 Range=0-150	0.2188

*Two-tailed Pearson correlation. Differences were considered statistically significant at $P < 0.05$.

1.2 Supplementary Figures

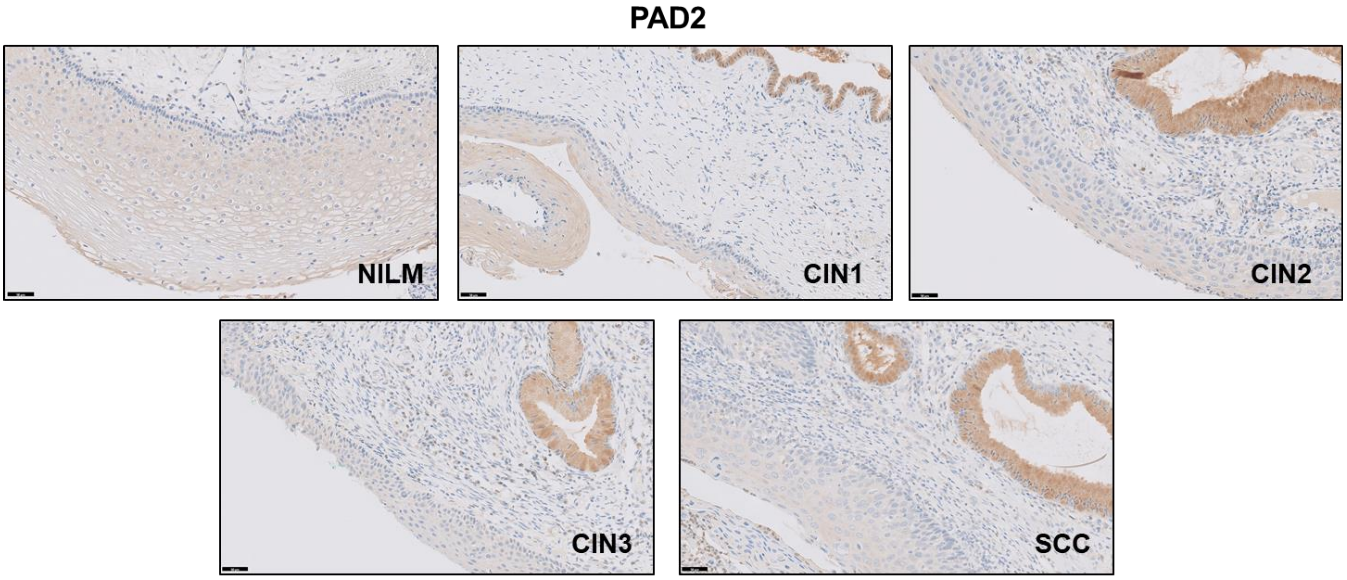

FIGURE S1. PAD2 expression in CINs and SCC. PAD2 immunohistochemical photomicrographs of representative biopsies of mucosa negative for intraepithelial lesion or malignancy (NILM) and

different stages of cervical carcinogenesis (CIN1, CIN2, CIN3, and SCC). Hematoxylin was used for counterstaining. Original magnification: 20X.

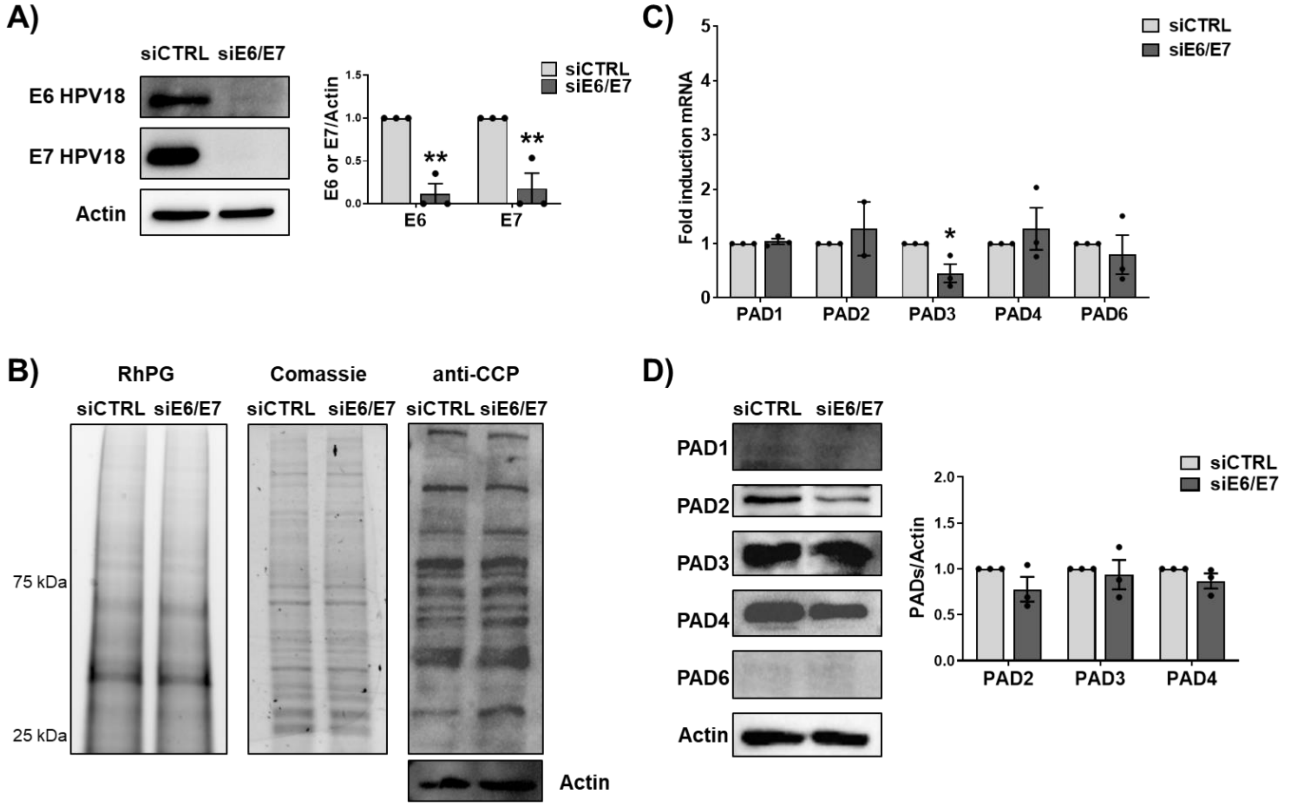

FIGURE S2: Citrullination analysis and PAD expression profile in HeLa cells. (**A**) HeLa cells were transfected with siRNA E6/E7 or siRNA CTRL and processed at 72 hours post-transfection. The efficiency of E6 and E7 protein depletion was assayed by Western blot analysis for E6 and E7 from HPV18, and Actin as a loading control. Values are expressed as means ± SEM (error bars) of three independent experiments. (**B**) Detection of citrullinated proteins in lysates obtained from HeLa transfected with siRNA E6/E7 or siRNA CTRL, at 72 hours post transfection. *Left panels:* lysates were exposed to an Rh-PG citrulline-specific probe and subjected to gel electrophoresis to detect total proteins. Equal loading was assessed by Coomassie blue staining. *Right panel:* the indicated samples were analyzed by western blotting, and citrullinated proteins were detected using anti-cyclic citrullinated peptide (CCP) antibody and actin as a loading control. (**C**) mRNA expression levels of PADI isoforms by RT-qPCR of HeLa transfected with siRNA E6/E7 or siRNA CTRL were normalized to the housekeeping gene GAPDH and expressed as mean fold change ± SEM over siRNA CTRL. (**D**) Western blot analysis of protein lysates from HeLa transfected with siRNA E6/E7 or siRNA CTRL using antibodies against PAD1, PAD2, PAD3, PAD4, PAD6, or Actin. One representative blot and predictive densitometric analysis is shown from three independent experiments. Values are expressed as mean fold change ± SEM normalized to Actin. Differences were considered statistically significant for P<0.05 (*, P<0.05; **, P<0.01, unpaired t-test).

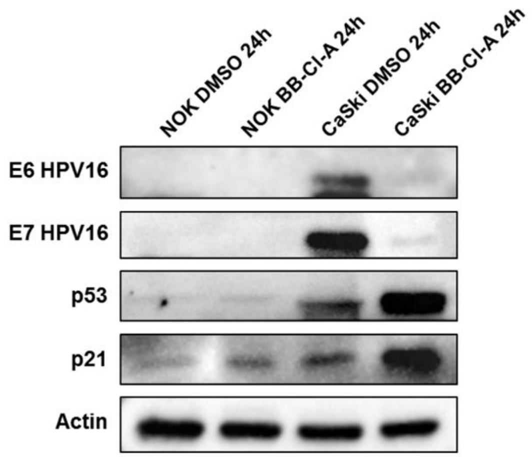

FIGURE S3: Effect of the pan-PAD inhibitor BB-Cl-amidine on HPV-negative normal oral keratinocytes (NOKs). Protein lysates from NOKs and CaSki cells treated with 3 µM BB-Cl-A were subjected to immunoblotting using antibodies against E6, E7, p53, p21, or Actin. One representative blot is shown.

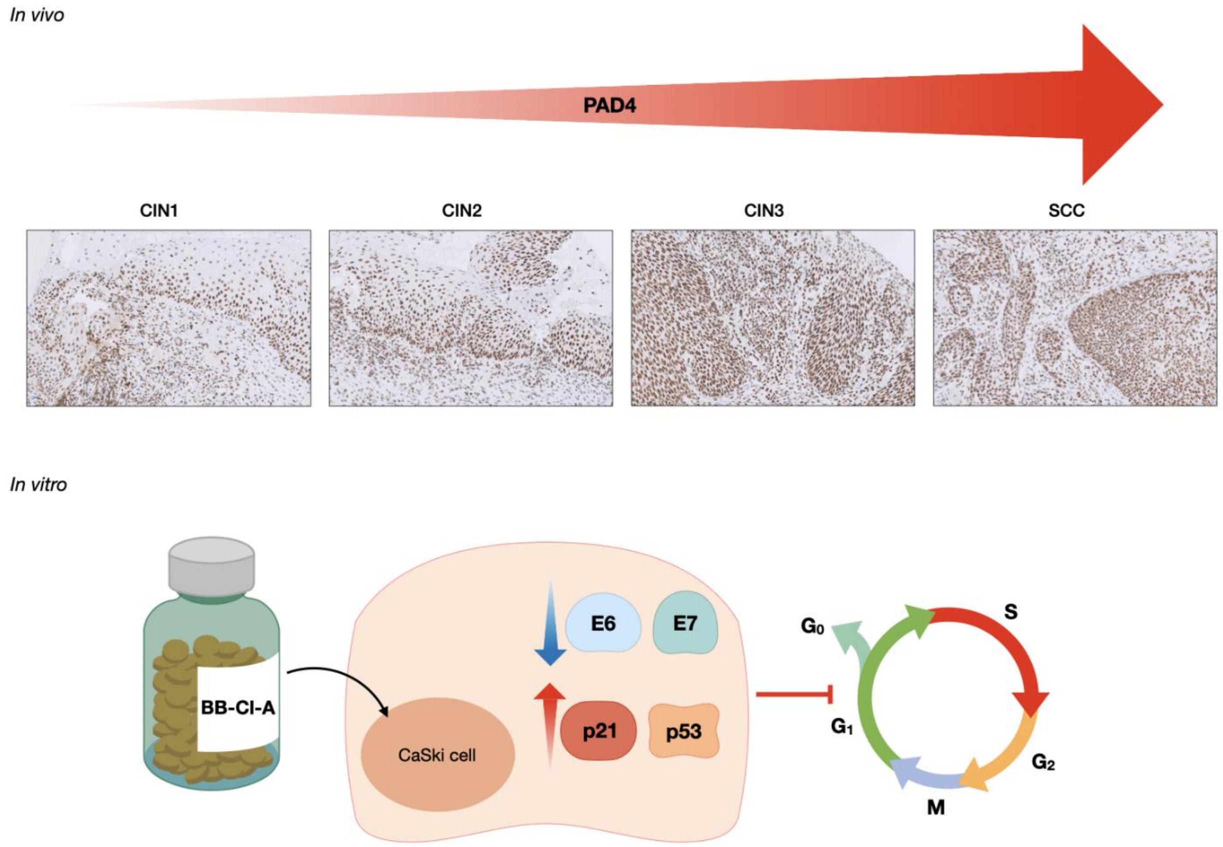

FIGURE S4: Model illustrating the interplay between PAD4 expression, cervical cancer progression, and the citrullination dynamics. PAD4 exhibits increased expression levels as lesions progress from low-grade (CIN1) to high-grade (CIN2, CIN3) cervical intraepithelial neoplasia, and invasive squamous cell carcinoma (SCC). In our CaSki *in vitro* model, the application of the pan-PAD inhibitor

BB-Cl-A results in the suppression of E6 and E7 HPV oncoprotein expression. Consequently, there is an upregulation of p53 and p21, leading to cell growth arrest and apoptosis.
